# The difference in leg lengths following total knee replacement for patients with severe osteoarthritic deformity

**DOI:** 10.1007/s00264-023-05948-x

**Published:** 2023-08-31

**Authors:** Mahmoud A. Hafez, Mohamed Mosa, Ahmed Abdelaal, Ahmed Moghny, Abdelrahman M. Makram

**Affiliations:** 1 The Orthopedic Department, Faculty of Medicine, October 6 University, Giza, Egypt; 2 The Orthopaedic Department, Faculty of Medicine, Al-Azhar University, Assiut, Egypt; 3 The Orthopaedic Department, Faculty of Medicine, Assiut University, Assiut, Egypt

**Keywords:** Leg length, Total knee replacement, Osteoarthritis

## Abstract

**Purpose:**

Increased height after total knee replacement surgery (TKR) may offer patients higher satisfaction as well as the quality of life. Therefore, in this paper, we aim to document the changes in leg length after TKR in patients with severe bilateral deformities.

**Methods:**

The data of 61 patients were collected from the Egyptian Community Arthroplasty Register; of them, 21 patients had unilateral TKR while 40 had bilateral simultaneous TKR. The patterns of changes in height of 101 osteoarthritic knees were followed up for 1 year after having TKR. All patients had standing leg X-rays, before and after surgery, to document the length of the femur and tibia before and after TKR. Correlations were assessed using the two-sample *t*-test.

**Results:**

The sample was mostly females (56/61, 91.8%). The distribution of the operated side was nearly equal (right knee was 47/101, 46.5%). The overall average leg length difference was 5.4 (SD = 2.3); for the unilateral group, the average was 4.6 (SD = 2.6); and for the bilateral group, the average was 5.6 (SD = 2.3), *p* = 0.119. We found that leg length may differ according to the varus deformity angle (*p* < 0.001) as well as fixed flexion deformity (*p* < 0.001).

**Conclusions:**

Leg length increased significantly 1 year after TKR. However, there is not enough evidence to suggest that the bilateral group had a greater height increase when compared to the unilateral group.

**Supplementary Information:**

The online version contains supplementary material available at 10.1007/s00264-023-05948-x.

## Introduction

Unilateral lower limb shortening leads to functional impairment such as difficulty walking or climbing. It is commonly associated with compensatory limb abnormalities and may lead to degenerative arthritis of the hip, knee, and/or lumbar spines [1]. It can be post-traumatic or developmental. Patients with unilateral lower limb shortening may have other deformities or soft tissue contractures that would influence their daily life activities [2, 3].

Leg length difference has been addressed in previous reviews as one of the results of severe knee arthritis where leg shortening is attributed to cartilage loss, varus, valgus, or flexion deformity. Leg length discrepancy is also the most potential explanation of lower back pain following knee osteoarthritis [4, 5]. Therefore, precise pre- and post-operative measurements of leg length and alignment are necessary to plan and evaluate the success of total knee replacement (TKR) as well as the range of motion, limb stability, and degree of deformity [6].

Consequently, not achieving an accepted leg length equalization after TKR is a major clinical problem and may lead to failure to regain normal walkability together with the existence of pain and discomfort [7]. Adjusting leg length would increase the patient’s satisfaction and physical activity after TKR surgery [8]. In other words, the leg length difference is one of the problems that may complicate TKR. However, this is detected in only a few cases that suffer from bilateral knee deformity and are treated unilaterally (one knee with the more severe arthritis) [9]. Surgeons who perform bilateral simultaneous TKR always consider correcting leg length as one of their treatment goals [8, 10].

And because literature is abundant regarding leg length issues in total hip replacement (THR) [11], surgeons can adjust leg length in hip replacement even in the absence of deformities, but this cannot be achieved in TKR. This is because surgeons can only restore normal length after correcting existing deformities during TKR. Moreover, the literature is still lacking clear-cut evidence about the different effects of unilateral versus bilateral TKR in leg length post-TKR. Subsequently, in this study, we aimed to document the changes in leg length after unilateral and bilateral TKR and to compare the results.

## Methods

### Study design

Before data collection, ethical approval was obtained from the Ethical Committee of our institution. The data described in this study were isolated from a large database of a clinical trial comparing TKR using conventional instruments versus patient-specific templating (PST) [12, 13]. Therefore, the reporting of this cross-sectional study was checked against the Strengthening the Reporting of Observational Studies in Epidemiology (STROBE) checklist [14], the version for cross-sectional studies (Supplementary Table 1). Ethical approval was acquired before the conduction of the study from the Hospital Ethical Committee. All participants agreed to be part of this study by signing a written consent form.

### Participants

A total number of 101 osteoarthritic knees of 61 patients were included in this study; of them, 21 patients had unilateral TKR while 40 had bilateral TKR. The data of the patients who performed TKR were collected prospectively from the Egyptian Community Arthroplasty Register (ECAR) [15]. Participants were only included if they had severe osteoarthritis with unilateral or bilateral deformities (varus, valgus, or flexion). All patients with bilateral deformities had bilateral simultaneous TKR. To limit other confounding factors, we only included patients who performed the surgery using the PST technique and excluded those who had their surgeries using conventional TKR systems.

### Imaging studies

Because the most commonly used imaging method for measuring leg length is digital full-leg radiographs in a standing position [16, 17], pre- and one year post-operative standing long-leg films were analyzed to determine the changes in leg length before and after TKR procedures. The radiographs were taken for initial screening of knee osteoarthritis and routine follow-up. The changes in the length of the femur and tibia before and after TKR were documented.

**Fig. 1:**
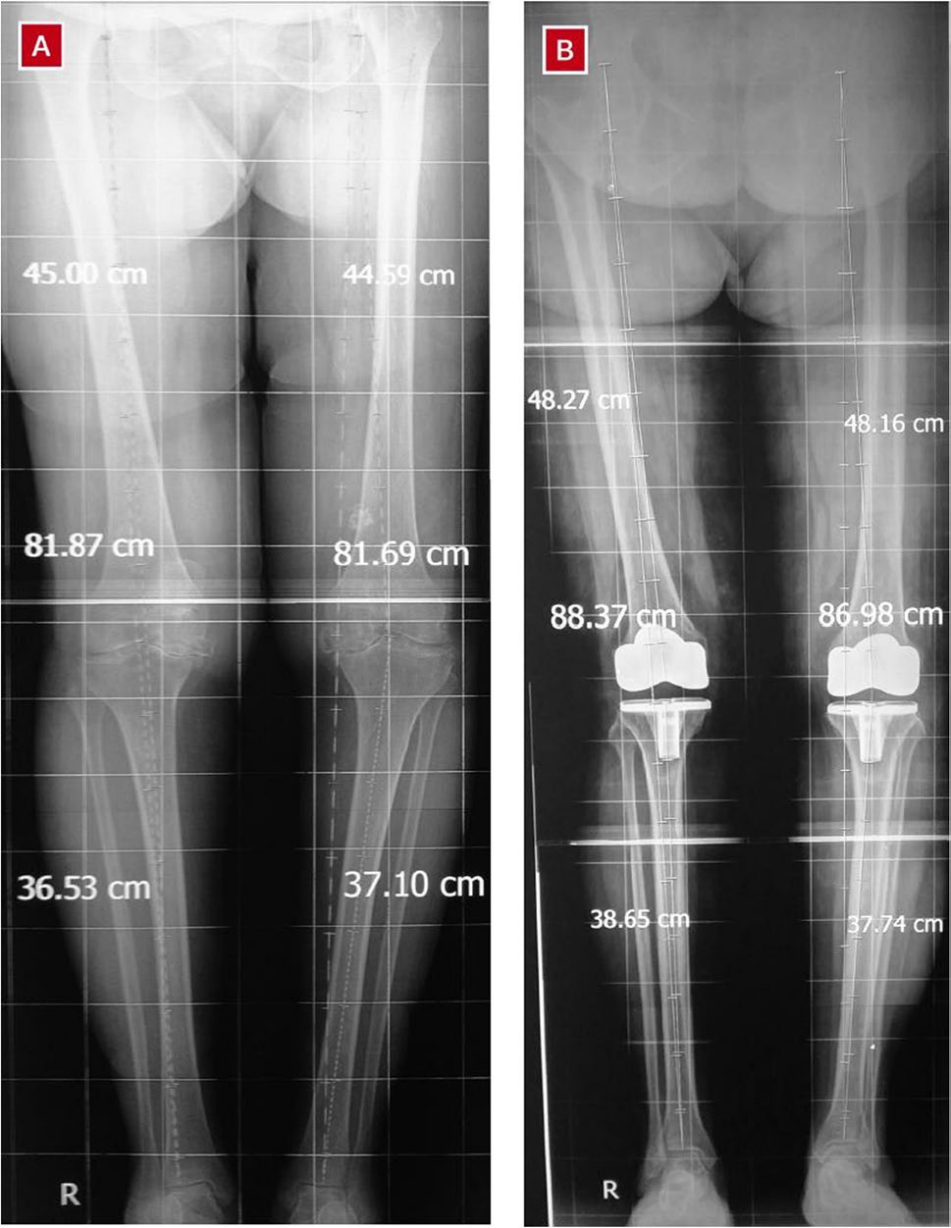
An illustration of how the measurements for leg length were taken before (**A**) and after (**B**) total knee replacement

We measured the length of the femur as the distance between the most proximal part of the femoral head and the centre of the intercondylar notch [18], while the length of the tibia was measured as the distance between the most proximal point of the sulcus (between the intercondylar eminence) and the tibiotalar joint line at the mediolateral centre of the ankle [19]; the total leg length was the sum of the lengths of the femur and tibia [20]. Figure 1 illustrates how the measurements were taken before and after TKR.

### Statistical analysis

Basic statistics were done by summarizing the variables into the mean and standard deviation (SD). Correlations were assessed using an independent two-sample Student’s *t*-test.

## Results

**Fig. 2:**
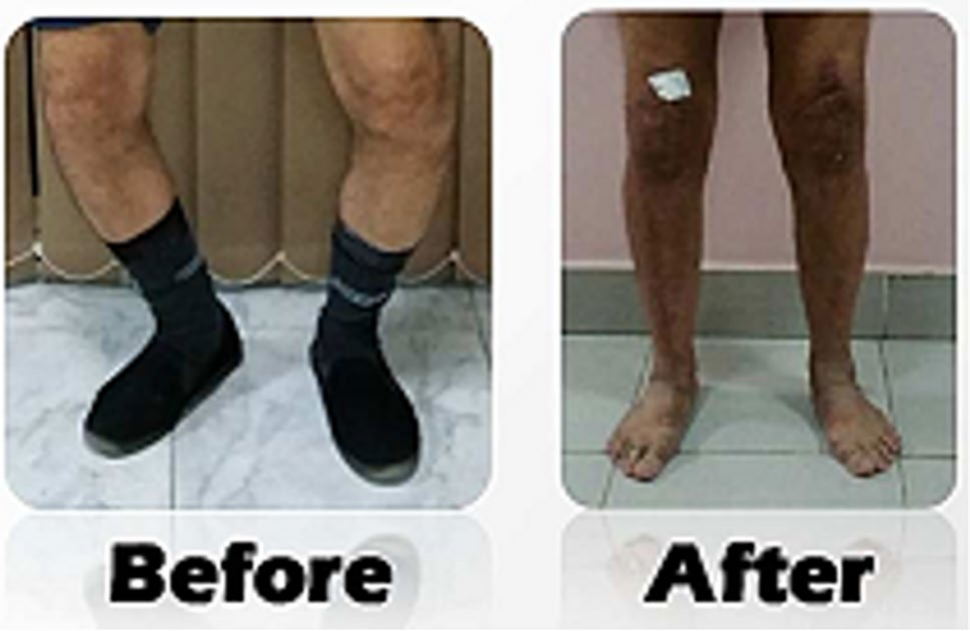
An illustration of how bilateral simultaneous total knee replacement has affected leg length in a patient

Of the included 61 patients, 56 were females (91.8%), and 93 out of the 101 (92.1%) of the knees also belonged to females. The distribution of the operated side was nearly equal (right knee was 47/101, 46.5%). The overall average leg length difference between the pre-operative and post-operative images was 5.4 (SD = 2.3); for the unilateral group, the average was 4.6 cm (SD = 2.6); and for the bilateral group, the average was 5.6 cm (SD = 2.3), *p* = 0.119. Further details about the basic characteristics of the included participants and their knees along with the leg length changes and pre-operative severity of deformities are presented in Table 1. We also found that leg length discrepancy may be much higher in people who had higher pre-operative varus deformity angle (*p* < 0.001) or fixed flexion deformity (*p* < 0.001) (Table 2). Figure 2 shows an image of a patient who received bilateral simultaneous TKR and how the operation affected leg length.

**Table 1:** Baseline characteristics of the included participants and their knees along with the leg length changes and pre-operative severity of deformities

	All participants	Unilateral TKR	Bilateral TKR
Total participants	61 (100%)	21 (34.4%)	40 (65.6%)
Male	5 (8.2%)	1 (1.6%)	4 (6.6%)
Female	56 (91.8%)	20 (32.8%)	36 (59.0%)
Total knees	101 (100%)	21 (20.8%)	80 (79.2%)
Male	9 (8.9%)	1 (1.0%)	8 (7.9%)
Female	92 (91.1%)	20 (19.8%)	72 (71.3%)
Right side	47 (46.5%)	7 (6.9%)	40 (39.6%)
Left side	54 (53.5%)	13 (12.9%)	40 (39.6%)
Leg length (cm)			
Pre-operative	76.3 (4.8), 64.9–91.7	76.9 (5.1), 70.2–91.7	76.1 (4.7), 64.9–89.0
Post-operative	81.6 (4.5), 69.6–94.7	81.5 (4.5), 73.9–94.7	81.7 (4.5), 69.6–93.1
Difference	5.4 (2.3), 0.2–11.1	4.6 (2.6), 0.2–8.7	5.6 (2.3), 0.8–11.1
Deformities (degrees)			
Varus	13.6 (6.8), 5.0–35.0	12.1 (6.0), 5.0–30.0	13.9 (7.0), 5.0–35.0
Fixed flexion	14.2 (12.0), 0.0–45.0	8.3 (9.9), 0.0–35.0	15.7 (12.1), 0.0–45.0

Categorical variables are presented in absolute numbers (percentages) while the continuous variables are presented in mean (standard deviation), range. Valgus deformity was only reported in one bilateral patient on the right side, and the deformity angle was 15°

**Table 2:** Factors affecting leg length in all participants, unilateral TKR, and bilateral TKR using Student’s *t*-test

	All participants (*N* = 61)	Unilateral TKR (*N* = 21, 34.4%)	Bilateral TKR (*N* = 40, 65.6%)
Varus deformity	*T*-value = − 11.47, *p*-value < 0.001*	*T*-value = − 5.25, *p*-value < 0.001*	*T*-value = − 10.24, *p*-value < 0.001*
Fixed flexion deformity	*T*-value = − 7.22, *p*-value < 0.001*	*T*-value = − 1.66, *p*-value = 0.053	*T*-value = − 7.36, *p*-value < 0.001*

^*^Reflects statistically significant results at the level of *p* < 0.05

## Discussion

In this work, we considered the correction of leg length difference while making TKR. Patients’ data were recorded, and radiographic findings (pre- and post-TKR) as well as post-operative functional outcomes were analyzed to determine the effect of TKR on leg length. We designed this work not only to highlight leg length changes following TKR but also to suggest TKR as a potential treatment option for patients with severe leg length differences due to severe osteoarthritis of the knee even at a young age.

It was observed that on ng unilateral TKR, the operated limb increases its length and improves its posture as a result of correcting its varus, valgus, or fixed flexion deformity. Several studies reported increased eccentric forces on the longer limb and thus the corrected limb may undergo a series of deteriorative conditions ending up with a worse outcome [21]. However, this is one reason that many orthopaedic surgeons prefer ng bilateral simultaneous TKR rather than unilateral or staged; thus, the patient would have both limbs managed in the same setting and rehabilitated at the same period to ensure minimal difference and proper walkability. This is in line with Vaidya et al. conclusion that leg length after unilateral TKR done for patients with bilateral knee osteoarthritis strongly influences the functional outcome and thus the opposing limb should be corrected as soon as possible [22].

Harvey et al. concluded that leg length difference is an important risk factor for the incidence of knee osteoarthritis similar to other factors such as obesity [23]. They also found that even an 0.5-cm leg length difference can be associated with increased odds of prevalent symptomatic osteoarthritis although physical examination may not provide accurate measurement of such amount of minor leg length difference. This minor leg length difference can be managed by shoe modification as an easy and cost-effective option with the possibility to correct the leg length difference over time.

Appropriate imaging for measuring leg length is vital for the proper management of the leg length difference. The accuracy of radiologists to provide proper assessment for more sophisticated radiological methods is an important factor for obtaining accurate results, although perceived leg length difference in some patients may be related to general dissatisfaction with the operation even without radiographic evidence [24]. This, together with the patient’s compliance and other radiological factors (e.g., time of exposure), are important factors that strengthen the results of leg length studies [25, 26]. In addition, reliability, accuracy, magnification, radiation dose, and ability to image the full extremity should be considered when assessing leg length [20]. However, it is reported that females and patients with poor functional outcomes are more likely to complain from leg length difference [24].

Increased post-operative leg length difference might affect functional outcomes of TKR; thus, treatment planning for cases of degenerative arthritis should consider the leg length issue, especially in unilateral TKR. For example, Kim et al. differentiated the functional outcomes of TKR when it is done for patients with leg length differences of more or less than 15 mm although a low correlation coefficient was found. Post-operative leg length difference was less improved following unilateral TKR than bilateral. In addition, patients who have higher values of leg length difference pre-operatively are more susceptible to a post-operative discrepancy. Thus, adjusting leg length should be one of the goals of TKR that the surgeon has to improve while planning surgery [27].

Another study by Goldstein et al. correlated body mass index, age, and mechanical knee alignment to leg length difference after TKR and found them not demonstrating any statistical difference. Another important finding of their work was that perceived leg length difference resolves within three months post-operatively [28].

Limitations of our study include the short follow-up period as well as the small sample size. Moreover, most cases that were included were females, preventing us from concluding the differences between both sexes. Still, this is the first study to report these outcomes in a group of Arab/Middle Eastern patients. Thirdly, the study had a main focus on leg length with TKR and did not consider other factors that may be related to limb shortening such as infections or slipped capital femoral epiphysis [29]. Lastly, we did not correlate the degree of deformities to changes in height.

## Conclusion

Both unilateral and bilateral TKR could increase the leg length of the operated limb(s) after surgery. However, there is not enough evidence to suggest that the bilateral group had a greater height increase when compared to the unilateral group. Regardless, we and other authors recommend that for patients with bilateral severe osteoarthritis and deformities, it is preferable to do bilateral simultaneous TKR to restore height and improve patients’ satisfaction. Doing a unilateral TKR in such cases will lead to leg length inequality and dissatisfaction. Future longitudinal studies are required to assess the differences in leg length post-TKR in both sexes and to investigate the predictors of better leg length outcomes.

### Supplementary Information

Below is the link to the electronic supplementary material.

Supplementary file1 (DOCX 40 KB)

## Data Availability

The data used in this study is not publicly available due to the difficulty in updating the ECAR data and making an online version of it. However, all data can be requested from the corresponding author on reasonable request (MAH: mhafez@msn.com).
